# Adherence to the Cancer Prevention Recommendations from World Cancer Research Fund/American Institute for Cancer Research After Cancer Diagnosis on Mortality in South Korea

**DOI:** 10.3390/nu16234049

**Published:** 2024-11-26

**Authors:** Donghyun Won, Jeeyoo Lee, Sooyoung Cho, Ji Yoon Baek, Daehee Kang, Aesun Shin

**Affiliations:** 1 Department of Preventive Medicine, Seoul National University College of Medicine, Seoul 03080, Republic of Korea; donghyun229@snu.ac.kr (D.W.);; 2 Integrated Major in Innovative Medical Science, Seoul National University Graduate School, Seoul 03080, Republic of Korea; 3 Genomic Medicine Institute, Medical Research Center, Seoul National University, Seoul 03080, Republic of Korea; 4 Cancer Research Institute, Seoul National University, Seoul 03080, Republic of Korea; 5 Interdisciplinary Program in Cancer Biology Major, Seoul National University College of Medicine, Seoul 03080, Republic of Korea

**Keywords:** cancer survivors, adherence, cancer prevention guidelines, physical activity

## Abstract

Background/Objectives: The World Cancer Research Fund/American Institute for Cancer Research recommends following the recommendations for cancer prevention even after cancer diagnosis. To provide evidence on the potential benefits of adherence on improved survival, we investigated the effects of post-diagnostic adherence to the recommendations regarding diet, physical activity, and body weight on all-cause mortality among Korean cancer survivors. Methods: Among the total number of cancer survivors (*n* = 173,195) recruited from 2004 to 2013 for the Health Examinees study, 5485 were selected for the analyses and classified by tertiles according to the adherence score. The Cox proportional hazards model was used to estimate the adjusted hazard ratios (HR) and their 95% confidence intervals (CI) of the adherence groups on all-cause mortality. Results: Although no clear association was observed overall during a mean follow-up of 10.1 (standard deviation = 3.0) years, reduced mortality was observed for the middle (HR = 0.74 [95% CI = 0.51–1.08]) and highest adherent group (0.66 [0.43–0.99]) in comparison to the lowest adherent group among long-term survivors (>5 years after cancer diagnosis). Conclusions: Among the cancer prevention recommendation items, “be physically active” and “limit consumption of fast foods” were inversely associated with mortality. Adhering to the WCRF/AICR cancer prevention recommendations may help improve the prognosis of long-term cancer survivors.

## 1. Introduction

Cancer is recognized as a major barrier to increasing life expectancy, posing a significant challenge to public health. The Global Cancer Observatory estimates that there were 20 million new cancer cases and nearly 9.7 million cancer-related deaths in 2022 [1], showing an increase in incidence but a decrease in mortality in comparison to 2020 [2]. Following this trend, the number of cancer survivors, individuals who have survived after being diagnosed with cancer at least once, has also increased [3], leading to great interest in cancer survivorship [4]. In South Korea, cancer accounted for 26.0% (317,655) of all deaths, and the number of prevalent cancer cases in Korea has exceeded 2 million since 2018 [5]. Several cancer prevention recommendations have been published [6,7,8]. World Cancer Research Fund/American Institute for Cancer Research (WCRF/AICR) recently updated cancer prevention recommendations and also advised cancer survivors to follow them after cancer diagnosis [9]. They recommend cancer-related health behaviors which encompass diet, nutrition, and physical activity [8,10]: (1) Be a healthy weight; (2) Be physically active; (3) Eat a diet rich in whole grains, vegetables, fruits, and beans; (4) Limit consumption of “fast foods” and other processed foods high in fat, starches, or sugars; (5) Limit consumption of red and processed meat; (6) Limit consumption of sugar-sweetened drinks; (7) Limit alcohol consumption; (8) Do not use supplements for cancer prevention; (9) For mothers; breastfeed your baby, if possible; (10) After a cancer diagnosis: follow the recommendations, if possible. The research group further developed a standardized scoring system that included appropriate criteria for each component to enable research applications [9].

Previous systematic reviews and meta-analyses found inconclusive results when investigating the prognostic effects of maintaining body mass index, doing physical activity, or consuming proper nutrients across different study designs, cancer types, stages, and sex [11,12,13,14]. In addition, it has been suggested that adherence to only a single healthy behavior may not be enough to improve survival, highlighting the importance of a comprehensive adherence to multiple healthy behaviors [15,16]. However, the previous literature only observed the advantages of healthy behaviors cumulatively for specific cancer types such as colorectal cancer [17,18], prostate cancer [19], and breast cancer [20]. No previous studies have considered all components of the recommendations in a comprehensive manner for overall cancer survival in a nation-wide population-based cohort.

To fill the gap in research, the current study aimed to evaluate the effects of a comprehensive adherence to the WCRF/AICR cancer prevention recommendations after cancer diagnosis on all-cause mortality among Korean cancer survivors. We specifically conducted the stratification analysis by survival time (<5 years and ≥5 years after cancer diagnosis).

## 2. Materials and Methods

The Health Examinees study (HEXA) is a part of the Korean Genome and Epidemiology Study (KoGES), which was established for epidemiological research investigating the etiology of diseases and causes of death with long-term follow-up. This large-scale population-based prospective closed cohort consisted of men and women aged 40 and over who visited training hospitals or health examination centers between 2004 and 2013 [21]. 

We defined cancer survivors using information from questionnaires on past disease history in line with the definition of cancer survivors from the National Comprehensive Cancer Network [3]. We used a self-reporting questionnaire to define the study population with diagnoses of stomach, liver, colorectum, breast, cervix, lung, thyroid, prostate, and bladder cancer. 

**Figure 1:**
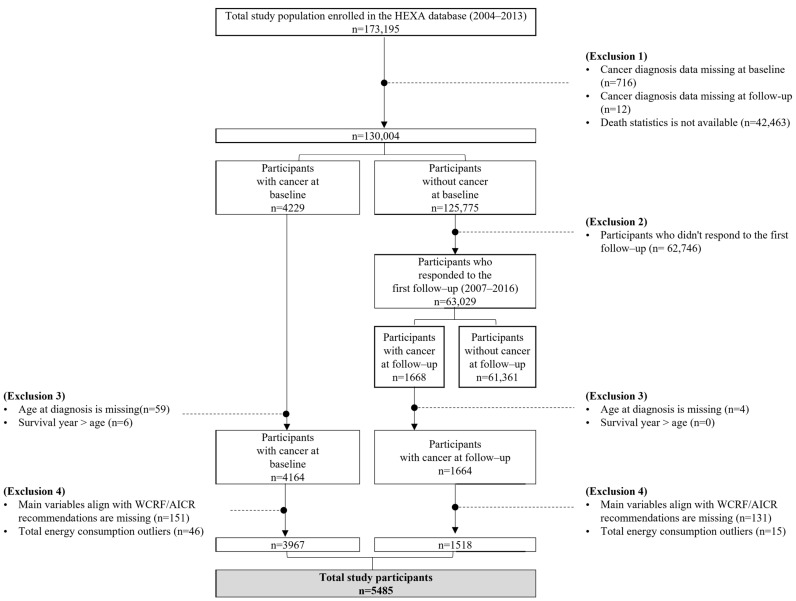
Flow chart of the study participants. HEXA, Health Examinees study; WCRF/AICR, World Cancer Research Fund/American Institute of Cancer Research.

Of the 173,195 participants in the HEXA cohort, we excluded 716 participants whose cancer history information was missing at baseline (*n* = 716) and follow-up (*n* = 12). An additional 42,463 participants who did not provide consent for the linkage to death statistics were also excluded. A total of 4229 participants were identified as cancer survivors in the baseline survey, and 1668 participants were further identified as cancer survivors in follow-up surveys. Participants whose age at diagnosis was missing (*n* = 63) and whose calculated survival years were longer than their reported age (*n* = 6) were excluded. We then excluded participants whose questionnaire data related to the WCRF/AICR recommendations were missing (*n* = 282). Participants whose total energy consumption exceeded the normal range (men: <800 kcal or >4000 kcal; women: <500 kcal and >3500 kcal) were excluded (*n* = 61) [22]. The total number of participants included in the final analyses was 5485; 3967 at baseline and 1518 at follow-up (Figure 1). 

The WCRF/AICR recommendations address cancer-related health behaviors encompassing diet, nutrition, and physical activity [8]. We used a 106-food item semi-quantitative food frequency questionnaire, which has been tested for validity and reliability, to evaluate adherence to diet and nutrition [23]. We also used a structured questionnaire to evaluate behaviors other than dietary habits and confounders [21]. All variables used to evaluate adherence were collected once at either baseline or follow-up, depending on whether they were collected before or after diagnosis. Sex, age, smoking status, years post diagnosis, cancer type, disease history of diabetes, and total energy intake were also collected once at the time of exposure measurement as post-diagnostic features. Information on education level and income level were only collected once at baseline regardless of cancer diagnosis status, assuming that those variables stay consistent [24].

We employed a standardized scoring system to evaluate adherence to the 2018 WCRF/AICR cancer prevention recommendations, with modifications as shown in Table S1 [10]. Participants were assigned a score for each component, ranging from 0 to 1, with 0 indicating non-adherence and 1 indicating full adherence. The criteria for BMI were adapted from those of the Korean Society for the Study of Obesity; 0.5 points for normal weight (18.5–22.9 kg/m^2^), 0.25 points for pre-obesity (23.0–24.9 kg/m^2^), and 0 points for others (<18.5 kg/m^2^ or ≥25.0 kg/m^2^) [25]. Waist circumference criteria were set at less than 90 cm for men or 85 cm for women, with 0.5 points given to participants who satisfied the criteria and 0 points for others [26]. The total points from both BMI and waist circumference criteria were summed for adherence classification: participants scoring below 0.25 classified as non-adherent, between 0.25 and 0.75 as moderately adherent, and above 0.75 as fully adherent. Physical activity was evaluated based on the total duration of moderate to vigorous physical activities per week, where 150 min and 75 min were used as the criteria as recommended. Total fiber intake was not available in our dataset; therefore, adherence to fruit and vegetable intake was scored on a scale from 0 to 1. Participants reporting more than 400 g of fruit and vegetable intake per day received 1 point, those consuming between 200 g and 400 g per day received 0.5 points, and all others received 0 points. Additionally, the percentage of total kcal from ultra-processed foods was substituted with total intake (g/day). Participants who consumed the least amount of ultra-processed foods per day received 1 point, those in the middle received 0.5 points, and those who consumed the most received 0 points. For sugar-sweetened drinks, the criteria were set at 0–250 g per day, and for ethanol consumption, either 0–28 g for men or 0–14 g for women. Participants who exceeded these limits received 0 points, those within the range received 0.5 points, and those who consumed 0 g received 1 point. Calculation methods and details of converting each food item into the recommended components are provided in Table S2. After calculating the points for each of the seven components, we added all points and assigned adherence scores ranging from 0 to 7 to each participant, where a higher score stands for higher adherence.

We divided the study population equally into three groups based on the adherence scores for statistical analyses, similar to the previous literature [27,28]. The lowest adherent group had scores below 3.75 (*n* = 1478, 27.0%), the middle adherence group consisted of individuals with scores between 3.75 and 4.75 (*n* = 2061, 37.6%), and those with scores higher than 4.75 (*n* = 1946, 35.5%) were defined as the highest adherence group.

All-cause mortality was ascertained through the linkage of the Korean Statistical Information Service-Microdata Integrated Service. The last follow-up date was 31 December 2021.

The basic and socioeconomic characteristics of participants were summarized using means and standard deviation (SD) for continuous variables, and the total number of study population and percentages for categorical variables. A chi-square test was conducted to identify features associated with the adherence scores using the absolute frequencies of the study population. We estimated the prognostic impact of adherence to WCRF/AICR cancer prevention recommendations on mortality using the Cox proportional hazards model. The results were presented using hazard ratios (HRs) and 95% confidence intervals (CIs), using age as the time scale [29]. Subjects exited at the age of death or on 31 December 2021. We also adjusted for potential confounding variables, including age, sex (men or women), years post diagnosis, smoking status (non-smokers, former smokers, current smokers, or missing), and diabetes (no, yes, or missing). Stratification by sex, years after cancer diagnosis, and cancer type was conducted. As a sensitivity analysis, we excluded patients who failed adherence within 1 year of initial cancer diagnosis, conducting the analysis using the Cox proportional hazard model.

Both SAS statistical software package version 9.4 (SAS Institute Inc., Cary, NC, USA) and R Core Team version 4.3.1 (2023, R Foundation for Statistical Computing, Vienna, Austria) were used for statistical analyses.

Informed consent was obtained on all participants, and ethical approval for this was obtained from Institutional Review Boards of Seoul National University Hospital in Seoul, Korea (no. E-2308-012-1454).

## 3. Results

Table 1 shows the basic and socioeconomic characteristics of cancer survivors according to their adherence to the WCRF/AICR cancer prevention recommendations. Participants with high adherence tended to be women, older, had lower education levels, had lower income levels, did not smoke, and reported lower energy consumption compared to those with low adherence. Adherence levels also differed between cancer types, with stomach cancer, breast cancer, and lung cancer survivors tending to adhere more compared to the other cancer survivors.

**Figure 2:**
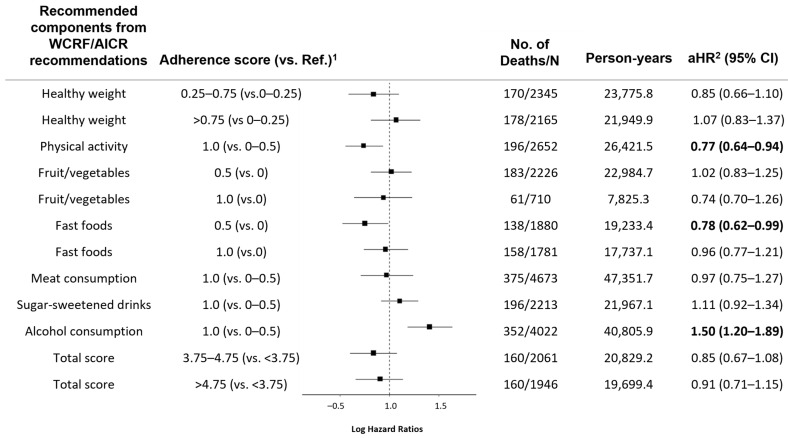
Effects of adherence to each component of the WCRF/AICR cancer prevention recommendations on all-cause mortality. WCRF/AICR, World Cancer Research Fund/American Institute of Cancer Research; N/No., the number of corresponding cases; Ref., reference; HR, Hazard Ratios; CI, Confidence Intervals. ^1^ Each recommended component was assigned a point ranging from 0.00 to 1.00, where 0.00 and 1.00 indicate non-adherence and full adherence, respectively. ^2^ Model was adjusted for age, sex (men, or women), smoking status (non-smokers, former smokers, current smokers, or missing), and diabetes (no, yes, or missing).

Figure 2 presents the effects of adherence to each recommended component on all-cause mortality. Participants who reported doing more than 150 min of moderate to vigorous physical activities per week were estimated to have a 23% reduction in all-cause mortality (0.77 [0.64–0.94]) compared to the others who reported shorter durations of physical activities. Lower all-cause mortality was also observed among those who consumed less fast foods (0.78 [0.62–0.99]) compared to those who were classified in the highest consumption group for ultra-processed foods. Non-drinkers had significantly increased mortality (1.50 [1.29–1.89]) compared to heavy or light drinkers. We could not find any significant association between adherence and all-cause mortality from the other recommended adherence components. Additionally, adherence status stratified by sex is shown in Figures S1 and S2.

The study population showed good adherence regarding meat intake and alcohol consumption, with more than 50% fully adhering to components. In contrast, adherence to fruit and vegetable intake was low, with only 14.6% (*n* = 216) of men and 12.3% (*n* = 494) of women meeting the criteria of full adherence. We observed a large difference (26.9%) in adherence to alcohol consumption between the sexes (men: 53.7%; women: 80.6%), with women being more likely to fully meet the optimal adherence criteria.

Table 2 and Table 3 present the combined effects of adherence to the cancer prevention recommendations on all-cause mortality among study populations, stratified by sex and years after cancer diagnosis, respectively. During a mean follow-up of 10.1 (SD = 3.0) years, we identified 1479 (27.0%) men with a mean age of 61.1 years (SD = 7.9) and 4006 (73.0%) women with a mean age of 55.4 years (SD = 7.9). Men in the middle (adjusted HR = 0.88 [95% CI = 0.65–1.21]) and highest adherent group (0.93 [0.68–1.29]) had reduced mortality compared to the lowest adherent group, but these results were not statistically significant (Table 2). We observed a significant linear decline in the association (*p* for trend < 0.05) between adherence and all-cause mortality, with reduced mortality for the middle (0.74 [0.51–1.08]) and highest adherent group (0.66 [0.43–0.99]), particularly among participants who survived more than 5 years after diagnosis (Table 3). Table S3 presents the results by cancer type, showing trends where higher adherence scores were related to decreased mortality among patients with stomach cancer, liver cancer, cervix cancer, and prostate cancer. However, these associations were not statistically significant.

Among the total number of cancer survivors included in the study (*n* = 5485), those whose survival year was less than a year (*n* = 1239) were excluded from the sensitivity analysis (Table S4). During a mean follow-up of 10.2 years (SD = 3.0), we found an inverse relationship between all-cause mortality and adherence to physical activity, consumption of fruit & vegetables, and red & processed meat. Meeting all adherence criteria for each component was associated with reduced all-cause mortality. However, statistical significance was not guaranteed.

## 4. Discussion

This study found a lowered mortality among cancer survivors who adhered to the WCRF/AICR cancer prevention recommendations after cancer diagnosis, particularly among cancer survivors with more than 5 years of survival time. Additionally, the current study observed a decrease in all-cause mortality among cancer survivors who engaged in more physical activity or reduced fast food intake after cancer diagnosis.

No studies have yet explored the prognostic impact of adherence to the WCRF/AICR cancer prevention recommendations among cancer survivors in an Asian population. Our findings are consistent with results from cohort studies conducted among the Western populations. The Moli-sani cohort study in Italy found that higher adherence to WCRF/AICR cancer prevention recommendations was associated with lower all-cause mortality among cancer survivors [30]. A cohort study in the United States found an inverse association between healthy lifestyles and mortality, with a high to moderate lifestyle score being associated with a lower risk of death (0.81 [072, 0.90] per 1 unit increase) [31]. However, more research is needed to establish this relationship among all cancer survivors as previous studies have mostly focused on specific cancer types, especially colorectal [17,18] and breast cancer [32]. Moreover, previous studies have not reported findings that support post-diagnosis adherence, despite the potential influence of survival duration on prognosis, taking into consideration the increasing number of long-term survivors [5].

The current study found inverse associations between the comprehensive adherence to the cancer prevention recommendations and all-cause mortality, which was highly dependent on survival time. These associations were only observed in participants who survived more than 5 years, which might be explained by the behavioral features of cancer survivors reported in the previous literature. A previous systematic review found that adherence rates depended on survival time; recent survivors (<5 years of survival time) showed relatively better adherence to multiple health behaviors than long term survivors (≥5 years of survival time) [16]. The study also suggested that motivation levels to change behaviors among cancer survivors might diminish over time [16]. Another study observed that cancer patients who survived more than 5 years had lower mortality, likely because they were able to overcome the challenges associated with poor adherence to positive health behaviors that often arise with prolonged survival time [33].

We further explored the association between each respective component and all-cause mortality to identify priorities among them when it comes to adherence. Decreased mortality was observed among cancer survivors who engaged in more physical activity or reduced fast food intake after cancer diagnosis. Previous studies have suggested the benefits of doing physical activity in reducing mortality, especially among colorectal [34] and breast cancer survivors [35]. Physical activity may directly reduce mortality by decreasing tumor hypoxia, increasing blood flow, and improving the delivery of anticancer therapies [36]. It could also indirectly contribute to lower mortality by enhancing other health outcomes, such as health-related quality of life and physical function [37]. Another cohort study suggested that a higher post-diagnostic intake of total ultra-processed foods in colorectal cancer survivors was associated with increased cancer-specific mortality [38]. The cumulative intake of ultra-processed foods may promote tumor proliferation and metastasis, potentially due to their high glycemic load and inflammatory potential [38,39]. However, we found protective effects of alcohol consumption in lowering all-cause mortality among study participants. Further research is warranted on these associations as results from previous studies have varied across cancer types (colorectal cancer [40]; stomach cancer [41]; breast cancer [42]; prostate cancer [43]).

This study has several strengths. As a large-scale cohort study, it is the first study to investigate the impact of comprehensive post-diagnosis adherence to cancer prevention recommendations on all-cause mortality among cancer survivors in South Korea. Second, the long follow-up period helped minimize the confounding effects of survival time through adjustment and stratification analysis. Quality of life and medical conditions can be influenced by survival time as long-term effects of treatment or premature aging emerge as time passes [44,45]. Survival time should be considered as a confounder in this survival analysis since it was associated with both prognosis and post-diagnosis adherence to cancer prevention recommendations [16].

Our study also had several limitations. First, we used self-reported cancer diagnosis information to define the study population, which may have led to selection bias and information bias due to the misclassification of cancer survivors and their cancer types. However, the sensitivity and positive predictive value were reported as 72.0% and 81.9% for overall cancer, suggesting that self-reported cancer information has proven reliability [46]. Second, potential bias due to cancer staging remains a limitation. It is unclear whether the observed reduction in mortality is attributable to the early diagnosis of cancer or due to the prognostic impact of adopting healthy behaviors. This suggests the need for further research that takes into consideration the cancer stage at diagnosis, either by adjusting for cancer stage in the model or estimating cause-specific deaths, particularly cancer-specific deaths, for the analysis. Third, measurement bias may still exist, as results of the measurement can vary across institutions. However, its impact on the study results was minimized by conducting the examinations through trained staff with standardized procedures to collect data [21].

## 5. Conclusions

In conclusion, our study results indicate that comprehensive adherence to the WCRF/AICR cancer prevention recommendations had effects on reducing all-cause mortality, exclusively among long-term cancer survivors with more than 5 years of survival time. In addition, we observed that ‘doing moderate to vigorous physical activity’ or ‘limiting consumption of “fast foods” and other processed foods high in fat, starches, or sugars’ might be priorities for reducing mortality when it comes to Korean cancer survivors. We suggest that cancer survivors comprehensively adhere to the cancer prevention recommendations [47].

## Figures and Tables

**Table 1:** Basic and socioeconomic characteristics of cancer survivors according to adherence to the WCRF/AICR cancer prevention recommendations, the Health Examinees study (2004–2013).

	WCRF/AICR Adherence (Score, *n*)			
	Lowest Adherence (≤3.75, *n* = 1478)	Middle Adherence (3.75–≤4.75, *n* = 2061)	Highest Adherence (>4.75, *n* = 1946)	*p* Value ^1^
	N (%)	N (%)	N (%)	
Sex				
Men	453 (30.6)	552 (26.8)	474 (24.4)	<0.001
Women	1025 (69.4)	1509 (73.2)	1472 (75.6)	
Age				
<50	375 (25.4)	407 (19.7)	305 (15.7)	<0.001
50–59	582 (39.4)	794 (38.5)	854 (43.9)	
60–69	443 (30.0)	740 (35.9)	668 (34.3)	
≥70	78 (5.3)	120 (5.8)	119 (6.1)	
Education level ^2^				
≤Elementary school	232 (15.7)	352 (17.1)	319 (16.4)	0.01
Middle school	219 (14.8)	370 (18.0)	375 (19.3)	
≥High school	1012 (68.5)	1326 (64.3)	1239 (63.7)	
Missing	15 (1.0)	13 (0.6)	13 (0.7)	
Income level (10^4^ Korean won)				
Low (<150)	286 (19.4)	452 (21.9)	433 (22.3)	0.05
Lower-middle (150–<300)	485 (32.8)	667 (32.4)	576 (29.6)	
Upper-middle (300–<400)	253 (17.1)	323 (15.7)	339 (17.4)	
High (≥400)	322 (21.8)	438 (21.3)	387 (19.9)	
Not measured	20 (1.4)	40 (1.9)	40 (2.1)	
Missing	112 (7.6)	141 (6.8)	171 (8.8)	
Smoking status				
Non-smokers	1077 (72.9)	1589 (77.1)	1582 (81.3)	<0.001
Former smokers	266 (18.0)	376 (18.2)	311 (16.0)	
Current smokers	132 (8.9)	93 (4.5)	49 (2.5)	
Missing	3 (0.2)	3 (0.1)	4 (0.2)	
Years post diagnosis ^3^				
<5	878 (59.4)	1213 (58.9)	1189 (61.1)	0.33
≥5	600 (40.6)	848 (41.1)	757 (38.9)	
Cancer type				
Stomach	193 (13.1)	300 (14.6)	298 (15.3)	<0.001
Liver	24 (1.6)	51 (2.5)	38 (2.0)	
Colorectum	112 (7.6)	141 (6.8)	159 (8.2)	
Breast	172 (11.6)	316 (15.3)	411 (21.1)	
Cervix	167 (11.3)	190 (9.2)	146 (7.5)	
Lung	28 (1.9)	49 (2.4)	62 (3.2)	
Thyroid	376 (25.4)	477 (23.1)	394 (20.2)	
Prostate	43 (2.9)	62 (3.0)	39 (2.0)	
Bladder	23 (1.6)	28 (1.4)	26 (1.3)	
Others	309 (20.9)	416 (20.2)	347 (17.8)	
Missing	31 (2.1)	31 (1.5)	26 (1.3)	
Disease history of diabetes				
Yes	106 (7.2)	141 (6.8)	162 (8.3)	0.13
No	1372 (92.8)	1920 (93.2)	1782 (91.6)	
Missing	0 (0.0)	0 (0.0)	2 (0.1)	
Total energy intake ^4^				
Lowest intake (tertile 1)	373 (25.2)	752 (36.5)	701 (36.0)	<0.001
Middle intake (tertile 2)	474 (32.1)	675 (32.8)	685 (35.2)	
Highest intake (tertile 3)	631 (42.7)	634 (30.8)	560 (28.8)	

Abbreviation: WCRF/AICR, World Cancer Research Fund/American Institute of Cancer Research; N (n), the number of corresponding cases. ^1^ *p*-values are the results from chi-square test. ^2^ ‘Below elementary school’ includes those who dropped out from middle school, while ‘below middle school’ also includes those who dropped out from high school. ^3^ Years post diagnosis was the time interval between cancer diagnosis and post-diagnosis adherence measurements. ^4^ Tertile 1 (men: <1514.86 kcal/day, women: <1397.71 kcal/day), Tertile 2 (men: 1514.86–<1870.36 kcal/day, women: 1397.71–<1755.33 kcal/day), and Tertile 3 (men: ≥1870.36 kcal/day, women: ≥1755.33 kcal/day).

**Table 2:** Association between the adherence to WCRF/AICR recommendations and all-cause mortality, stratified by sex (Hazard ratios (HRs) and 95% Confidence Intervals (CIs)).

	Men (*n* = 1479)					Women (*n* = 4006)				
WCRF/AICR Adherence (Score)	N/No. Deaths	Person-Years	HR (95% CI)	aHR ^1^ (95% CI)	*p* Trend	N/No. Deaths	Person-Years	HR ^1^ (95% CI)	aHR ^2^ (95% CI)	*p* Trend
Lowest adherent (≤3.75)	453/77	4302.5	Ref.	Ref.	0.68	1025/46	10,701.6	Ref.	Ref.	0.29
Middle adherent (3.75–≤4.75)	552/87	5202.0	0.86 (0.63–1.17)	0.88 (0.65–1.21)		1509/73	15,627.1	1.00 (0.69–1.44)	1.01 (0.70–1.47)	
Highest adherent (>4.75)	474/78	4526.0	0.85 (0.62–1.17)	0.93 (0.68–1.29)		1472/82	15,173.4	1.17 (0.81–1.68)	1.20 (0.83–1.73)	

Abbreviation: WCRF/AICR, World Cancer Research Fund/American Institute of Cancer Research; N/No., the number of corresponding cases; Ref., reference; HR, Hazard Ratios; aHR, adjusted Hazard Ratios; CI, Confidence Intervals. ^1^ Model was not adjusted. ^2^ Model was adjusted for age, years post diagnosis, smoking status (non-smokers, former smokers, current smokers, or missing), and diabetes (no, yes, or missing).

**Table 3:** Association between the adherence to WCRF/AICR recommendations and all-cause mortality, stratified by years post cancer diagnosis (Hazard ratios (HRs) and 95% Confidence Intervals (CIs)).

	Years Post Diagnosis < 5 (*n* = 3280)					Years Post Diagnosis ≥ 5 (*n* = 2205)				
WCRF/AICR Adherence (Score)	N/No. Deaths	Person-Years	HR ^1^ (95% CI)	aHR ^2^ (95% CI)	*p* Trend	N/No. Deaths	Person-Years	HR ^1^ (95% CI)	aHR ^2^ (95% CI)	*p* Trend
Lowest adherent (≤3.75)	878/73	8566.3	Ref.	Ref.	0.08	600/50	6437.9	Ref.	Ref.	0.05
Middle adherent (3.75–≤4.75)	1213/98	11,544.2	0.96 (0.71–1.30)	1.06 (0.78–1.43)		848/62	9285.0	0.69 (0.48–1.01)	0.74 (0.51–1.08)	
Highest adherent (>4.75)	1189/116	11,474.6	1.13 (0.84–1.52)	1.29 (0.96–1.74)		757/44	8224.8	0.58 (0.38–0.86)	0.66 (0.43–0.99)	

Abbreviation: WCRF/AICR, World Cancer Research Fund/American Institute of Cancer Research; N/No., the number of corresponding cases; Ref., reference; HR, Hazard Ratios; aHR, adjusted Hazard Ratios; CI, Confidence Intervals. ^1^ Model was not adjusted. ^2^ Model was adjusted for age, sex (men, or women), smoking status (non-smokers, former smokers, current smokers, or missing), and diabetes (no, yes, or missing).

## Data Availability

Data is unavailable due to privacy or ethical restrictions.

## Supplementary Materials

The following supporting information can be downloaded at: https://www.mdpi.com/article/10.3390/nu16234049/s1, Figure S1: Adherence proportions of WCRF/AICR cancer prevention recommendations among men; Figure S2: Adherence proportions of WCRF/AICR cancer prevention recommendations among women; Table S1: Adaptation of standardized scoring system on evaluating the adherence to WCRF/AICR cancer prevention recommendations; Table S2: Detailed calculation formula of the adherence scores; Table S3: Association between adherence to WCRF/AICR recommendations and all-cause mortality, stratified by cancer type (Hazard ratios (HRs) and 95% Confidence Intervals (CIs)); Table S4: Association between adherence to WCRF/AICR recommendations and all-cause mortality among study population who survived more than a year (Hazard ratios (HRs) and 95% Confidence Intervals (CIs)).
