# Supplementary material for: Adherence to the Cancer Prevention Recommendations from World Cancer Research Fund/American Institute for Cancer Research After Cancer Diagnosis on Mortality in South Korea
Source: Nutrients. 2024 Nov 26;16(23):4049. doi: 10.3390/nu16234049 (PMC11643377; doi:10.3390/nu16234049)
Supplement: Supplementary file 1 [file nutrients-16-04049-s001.zip › nutrients-3314104-supplementary.pdf]

Supplements

Table S1. Adaptation of standardized scoring system on evaluating adherence to WCRF/AICR cancer prevention recommendations

Recommendations	Operationalization of Recommendations	Adaptation	Points	
1. Be a healthy weight	**BMI (kg/m²)**			
	18.5-24.9	18.5-22.9	0.5	
	25.0-29.9	23.0-24.9	0.25	
	<18.5 or ≥30.0	<18.5 or ≥25.0	0	
	**Waist circumference (cm)**			
	Men:	Men:		
	<94	<90	0.5	0.5
	94-<102		0.25	
	≥102	≥90	0	0
	Women:	Women:		
	<80	<85	0.5	0.5
	80-<88		0.25	
	≥88	≥85	0	0
2. Be physically active	**Total moderate to vigorous physical activity (minutes/week)**			
	≥150		1	
	75-<150		0.5	
	<75		0	
3. Eat a diet rich in whole grains, vegetables, fruits, and beans	**Fruits and vegetables (g/day)**			
	≥400		0.5	1
	200-<400		0.25	0.5
	<200		0	0
	**Total fiber (g/day)**			
	≥30	Not available		
	15-<30			
	<30			
4. Limit consumption of "fast foods" and other processed foods high in fat, starches, or sugars	**Percent of total kcal from ultra-processed foods**	**Percent of total intake (g/day) from ultra-processed foods**		
	Tertile 1	Tertile 1 (Men: <10, Women: <7)	1	
	Tertile 2	Tertile 2 (Men: 10-≤31.71, Women: 7-≤26.29)	0.5	
	Tertile 3	Tertile 3 (Men: ≥31.71, Women: ≥26.29)	0	
5. Limit consumption of red and processed meat	**Total red meat (g/week) and processed meat (g/week)**			
	Red meat <500 (<18 oz) and processed meat <21 (<0.75 oz)		1	
	Red meat <500 (<18 oz) and processed meat 21-<100 (0.75 oz-<3 oz)		0.5	
	Red meat >500 (>18 oz) or processed meat ≥100 (≥3 oz)		0	
6. Limit consumption of sugar-sweetened drinks	**Total sugar-sweetened drinks (g/day)**			
	0		1	
	>0-≤250 (8.5 oz)		0.5	
	>250 (>8.5 oz)		0	
7. Limit alcohol consumption	**Total ethanol (g/day)**			
	Men:			
	0		1	
	>0-≤28 (2 drinks)		0.5	
	>28 (2 drinks)		0	

Recommendations	Operationalization of Recommendations	Adaptation	Points
	Women:<br>0<br>>0-≤14 (1 drink)<br>>14 (1 drink)		 1<br>0.5<br>0
**8. Do not use supplements for cancer prevention**	Not included in the scoring logic		
**9. For mothers: breastfeed your baby, if you can**	Not included in the scoring logic		
**10. After a cancer diagnosis: follow our Recommendations, if you can**	Not included in the scoring logic		

Abbreviation: WCRF/AICR, World Cancer Research Fund/American Institute of Cancer Research.

Table S2. Detailed calculation formula of the adherence scores

Recommendations	Items (unit or category)	Calculation formula
**1. Be a healthy weight**		
	• BMI (kg/m^2)<br>• Waist circumference (cm)	
**2. Be physically active**		
	• Average duration of exercising per occasion (minutes)<br>• Frequency of exercising per week (1-2 times = 1.5 times, 3-4 times = 3.5 times, 5-6 times = 5.5 times, everyday = 7 days)	Total moderate to vigorous physical activity (minutes/week)<br>= average duration (minutes) × frequency per week
**3. Eat a diet rich in whole grains, vegetables, fruits, and beans**		
	• Food consumptions (g/day)	Total consumptions of following items per day<br>= Cabbage Kimchi + Radish Kimchi/Water Kimchi + Radish Water Kimchi/Dongchimi + Other Kimchi + Pickled Vegetables + Radish Soup/Stew/Pickled Radish + Cabbage Soup + Spinach + Lettuce + Perilla Leaves + Vegetable Wraps/Salad + Other Green Vegetables + Dodder/Doraji + Bean Sprouts/Mung Bean Sprouts + Fernbrake/Sweet Potato Stems/Taros + Enoki Mushrooms + Other Mushrooms + Chili Leaves/Chamnamul/Wild Aster + Crown Daisy/Chives/Parsley + Cucumber + Carrot/Carrot Juice + Onion + Green Pepper + Zucchini + Winter Melon/Pumpkin/Juice from Winter Melon/Pumpkin
**4. Limit consumption of "fast foods" and other processed foods high in fat, starches, or sugars**		
	• Food consumptions (g/day)	Total consumptions of following items per day<br>= Ramen + Corn Flakes + Bread/Sandwich/Toast + Jam/Honey/Butter/Margarine (for spreading on bread) + Bread Roll/Sweet Roll/Full Bread + Other Breads + Cake/Chocolate Pie + Pizza/Hamburger + Cookie/Cracker/Snack + Candy/Chocolate + Ice Cream
**5. Limit consumption of red and processed meat**		
	• Food consumptions (g/day)	Total consumptions of following items per week<br>• Red meat: {Pork Belly + Grilled/Stir-fried/Spicy Stir-fried Pork/Pork Jjim (Boiled pork, Braised Pork Belly, Pig's Feet) + Offal (Internal organs, Tripe, Blood Sausage) + Steak/Beef Steak (Short ribs, Sirloin, Tenderloin, Bulgogi) + Dog Meat + Soup (Ox bone soup, Beef bone soup, short rib soup, Beef tendon soup, etc.) + Stew (Beef stew, Spicy beef soup, etc.)} ×7

Recommendations	Items (unit or category)	Calculation formula
		• Processed meat: {Processed Meat (Ham, Sausage)} ×7
**6. Limit consumption of sugar-sweetened drinks**	• Food consumptions (g/day)	Total consumptions of following items per day<br>= Carbonated Drinks (Cola, Cider) + Other Beverages (Yuja Tea, Plum Tea, Aloe Vera Juice, Persimmon Punch, Ginseng Tea, Sikhye (Sweet Rice Drink), Jujube Tea, Ssanghwa Tea, etc.)
**7. Limit alcohol consumption**	• Frequency of alcohol drinking (never = 0, once a month = 12/365, 2-3 times per month = 30/365, once a week = 48/365, 2-3 times per week = 120/365, 4-6 times per week = 240/365, once a day = 336/365, more than twice a day = 672/365)<br>• Average amount of alcohol drinking per occasion (the size of the glass varies depending on the type of alcohol)	• Total intake of ethanol (g/day)<br>= average amount of alcohol drinking × 0.785 × alcohol by volume<br>• Alcohol consumption<br>= total intake of ethanol (g/day) × frequency/day

Table S3. Association between adherence to WCRF/AICR recommendations and all-cause mortality, stratified by cancer type (Hazard ratios (HRs) and 95% Confidence Intervals (CIs))

	WCRF/AICR adherence (score)			
	Lowest adherent (≤3.75)	Middle adherent (3.75-≤4.75)	Highest adherent (>4.75)	P trend
**Stomach Cancer (n = 791)**				
N	193	300	298	
No. of deaths	20	28	25	
Person-years	1,956.0	3,002.6	3,125.0	
HR[1] (95% CI)	Ref.	0.91 (0.51-1.62)	0.70 (0.39-1.27)	0.23
aHR[2] (95% CI)	Ref.	0.95 (0.53-1.69)	0.78 (0.43-1.43)	0.41
**Liver Cancer (n = 113)**				
N	24	51	38	
No. of deaths	11	18	11	
Person-years	169.4	385.7	310.8	
HR[1] (95% CI)	Ref.	0.83 (0.38-1.80)	0.69 (0.29-1.63)	0.40
aHR[2] (95% CI)	Ref.	0.97 (0.43-2.19)	0.73 (0.30-1.78)	0.47
**Colorectal Cancer (n = 412)**				
N	112	141	159	
No. of deaths	9	15	17	
Person-years	1,135.3	1,378.3	1,615.0	
HR[1] (95% CI)	Ref.	1.24 (0.54-2.84)	1.29 (0.57-2.89)	0.56
aHR[2] (95% CI)	Ref.	1.57 (0.66-3.73)	1.51 (0.65-3.52)	0.37
**Breast Cancer (n = 899)**				
N	172	316	411	
No. of deaths	11	17	31	
Person-years	1,768.7	3,311.9	4,236.4	
HR[1] (95% CI)	Ref.	0.70 (0.33-1.52)	1.10 (0.55-2.21)	0.47
aHR[2] (95% CI)	Ref.	0.71 (0.32-1.56)	1.07 (0.53-2.15)	0.56
**Cervix Cancer (n = 503)[3]**				
N	167	190	146	
No. of deaths	7	7	5	
Person-years	1,876.7	2,153.2	1,606.5	
HR[1] (95% CI)	Ref.	0.93 (0.32-2.71)	0.78 (0.24-2.50)	0.67
aHR[2] (95% CI)	Ref.	0.93 (0.31-2.79)	0.81 (0.24-2.73)	0.74
**Lung Cancer (n = 139)**				

WCRF/AICR adherence (score)				
	**Lowest adherent (≤3.75)**	**Middle adherent (3.75-≤4.75)**	**Highest adherent (>4.75)**	**P trend**
N	28	49	62	
No. of deaths	8	16	21	
Person-years	223.6	403.3	470.7	
HR[1] (95% CI)	Ref.	1.19 (0.50-2.79)	1.25 (0.55-2.85)	0.61
aHR[2] (95% CI)	Ref.	1.20 (0.50-2.88)	1.35 (0.58-3.14)	0.48
**Thyroid Cancer (n = 1,247)**				
N	376	477	394	
No. of deaths	8	9	4	
Person-years	3,610.5	4,678.9	3,854.6	
HR[1] (95% CI)	Ref.	0.73 (0.28-1.91)	0.42 (0.13-1.40)	0.15
aHR[2] (95% CI)	Ref.	1.06 (0.39-2.91)	0.55 (0.16-1.88)	0.36
**Prostate Cancer (n = 144)[3]**				
N	43	62	39	
No. of deaths	6	6	4	
Person-years	378.4	553.1	356.9	
HR[1] (95% CI)	Ref.	0.64 (0.19-2.09)	0.68 (0.18-2.57)	0.53
aHR[2] (95% CI)	Ref.	0.77 (0.22-2.63)	0.72 (0.18-2.88)	0.63
**Bladder Cancer (n = 77)**				
N	23	28	26	
No. of deaths	5	4	5	
Person-years	226.5	271.4	248.3	
Crude HR (95% CI)	Ref.	0.77 (0.20-2.90)	0.82 (0.23-2.85)	0.76
Model 2 HR (95% CI)2	Ref.	0.61 (0.15-2.53)	0.81 (0.22-2.91)	0.75

Abbreviation: WCRF/AICR, World Cancer Research Fund/American Institute of Cancer Research; N/No., the number of corresponding cases; Ref., reference; HR, Hazard Ratios; aHR, adjusted Hazard Ratios; CI, Confidence Intervals. [1] Model was not adjusted. [2] Model was adjusted for age, years post diagnosis, smoking status (non-smokers, former smokers, current smokers, or missing), and diabetes (no, yes, or missing). [3] Sex was not adjusted among cervix cancer survivors and prostate cancer survivors.

Table S4. Association between adherence to WCRF/AICR recommendations and all-cause mortality among study population who survived more than a year (Hazard ratios (HRs) and 95% Confidence Intervals (CIs))

WCRF/AICR adherence (criteria)	No. of deaths / N (Total N = 4,246)	Person-years	aHR (95% CI)[1]	*P* value
**1. Be a healthy weight (summed point of bmi and waist circumference)**				
Non (≤ 0.25)	71 / 734	7,456.5	Ref.	
Moderate (0.25-≤0.75)	123 / 1,833	18,823.8	0.79 (0.59-1.06)	0.12
Full (>0.75)	133 / 1,679	17,196.7	1.01 (0.76-1.36)	0.93
**2. Be physically active (total moderate to vigorous physical activity, min/wk)**				
Non (<75) to Moderate (75-<150)	181 / 2,214	23,051.5	Ref.	
Full (≥150)	146 / 2,032	20,425.5	0.84 (0.67-1.05)	0.12
**3. Eat a diet rich in whole grains, vegetables, fruits, and beans (fruits and vegetables consumption, g/day)**				
Non (<200)	147 / 1,948	19,904.0	Ref.	
Moderate (200-<400)	131 / 1,721	17,985.2	0.94 (0.74-1.12)	0.60
Full (≥400)	49 / 577	6,397.8	0.92 (0.67-1.28)	0.62
**4. Limit consumption of "fast foods" and other processed foods in fat, starches, or sugars (percent of total intake of ultra-processed foods divided into tertile, g/day)**				
Non (m: ≥31.71, w: ≥26.29)	105 / 1,424	14,718.0	Ref.	
Moderate (m: 10-<31.71, w: 7-<26.29)	110 / 1,456	14,979.9	0.89 (0.68-1.17)	0.39
Full (m: <10, w: <7)	112 / 1,366	13,779.1	0.95 (0.72-1.25)	0.71
**5. Limit consumption of red and processed meat (total red meat and processed meat consumption, g/wk)**				
Non (r: ≥500 or p: ≥100) to Moderate (r: <500 and p: 21-<100)	52 / 624	6,373.7	Ref.	
Full (r: <500 and p: <21)	275 / 3,622	37,103.3	0.92 (0.68-1.24)	0.56
**6. Limit consumption of sugar-sweetened drinks (total sugar-sweetened drinks, g/day)**				
Non (>250) to Moderate (>0-250)	185 / 2,554	26,473.8	Ref.	
Full (0)	142 / 1,692	17,003.2	1.09 (0.87-1.35)	0.46
**7. Limit alcohol consumption (total ethanol, g/day)**				
Non (m: >28, w:>14) to Moderate (m:>0-28, w:>0-14)	92 / 1,163	11,821.1	Ref.	
Full (0 for both sex)	235 / 3,083	31,655.9	1.42 (1.09-1.84)	0.01
**Total Adherence (Sum of the scores)**				
Lowest Adherent Group (<3.75)	90 / 1,145	11,789.58	Ref.	
Middle Adherent Group (3.75-≤4.75)	124 / 1,617	16,500.06	0.96 (0.73-1.26)	0.75
Highest Adherent Group (>4.75)	113 / 1,484	15,187.34	0.97 (0.73-1.29)	0.85

Abbreviation: WCRF/AICR, World Cancer Research Fund/American Institute of Cancer Research; N/No., the number of corresponding cases; Ref., reference; HR, Hazard Ratios; aHR, adjusted Hazard Ratios; CI, Confidence Intervals. [1] Model was adjusted for age, years post diagnosis, smoking status (non-smokers, former smokers, current smokers, or missing), and diabetes (no, yes, or missing).

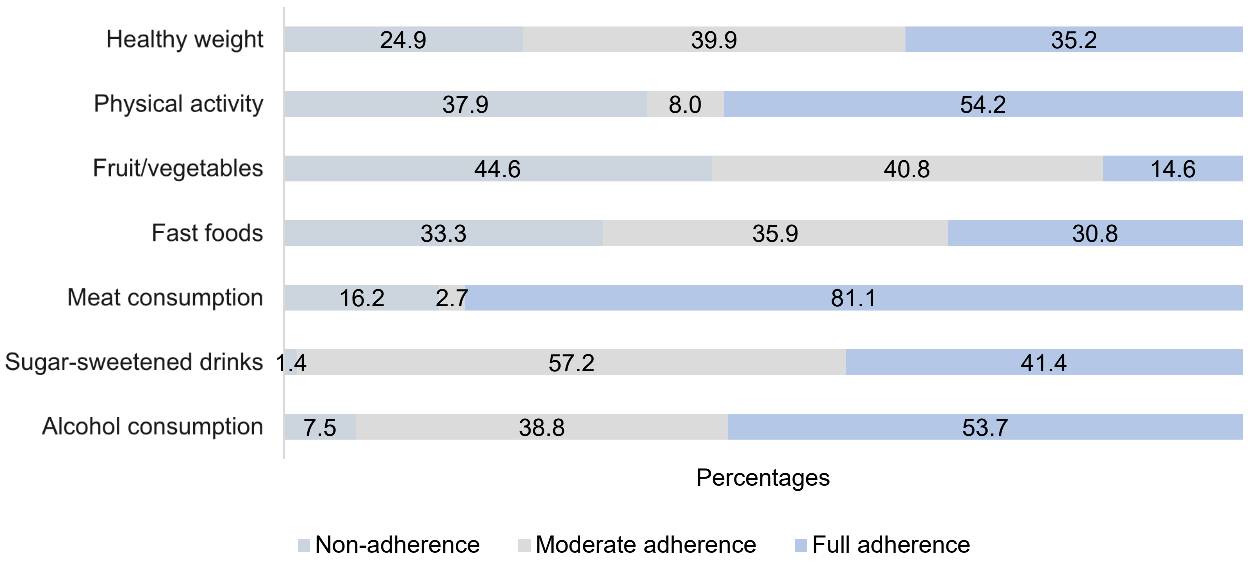

Figure S1. Adherence proportions of WCRF/AICR cancer prevention recommendations among men

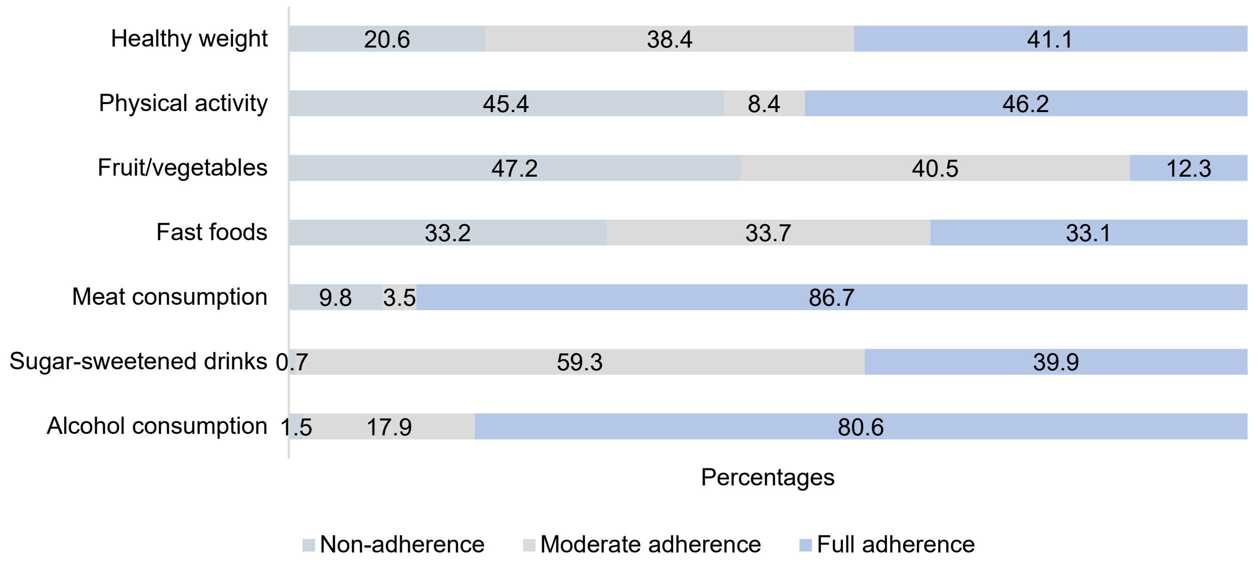

Figure S2. Adherence proportions of WCRF/AICR cancer prevention recommendations among women
